# Feasibility and initial assessment of a holistic neuropsychological day program for vocational rehabilitation following non-central nervous system cancer

**DOI:** 10.3389/fpsyg.2025.1415038

**Published:** 2025-06-13

**Authors:** Ayala Bloch, Limor Sharoni, Tal Shany-Ur, Sari Maril, Daniella Margalit

**Affiliations:** ^1^Department of Psychology, Ariel University, Ariel, Israel; ^2^The National Institute of Neuropsychological Rehabilitation, Tel Aviv, Israel; ^3^Department of Psychology, The Hebrew University of Jerusalem, Jerusalem, Israel

**Keywords:** cancer, CRCI, neuropsychology, vocational rehabilitation, fear of cancer recurrence, oncology, psycho-oncology

## Abstract

**Objectives:**

Neuropsychological difficulties are common following non-central nervous system (CNS) cancer. Five years after treatment, up to 40% of survivors still report deficits, supported by neuropsychological tests and MRI findings. As these deficits can pose significant difficulties in finding and maintaining employment, we developed a novel vocational rehabilitation day program for non-CNS cancer survivors. The 6-month program included individual and group interventions addressing cognitive and emotional difficulties, healthy lifestyle, and job placement. In a non-randomized controlled study, we compared changes in employment status and in cognitive and emotional variables, before and after program participation.

**Methods:**

46 individuals in the rehabilitation group were tested before and after participation in the program. Inclusion criteria were completion of treatment for non-CNS cancer, unemployment, and cognitive deficits shown in a neuropsychological assessment before the beginning of the program. They were compared to a control group of 15 participants who met the same inclusion criteria and were tested upon recruitment and 6 months later. Measures included employment status, a computerized cognitive test battery, and questionnaires assessing the emotional variables depression, anxiety, and fear of cancer recurrence.

**Results:**

The emotional variables showed greater improvement in the rehabilitation group than in the control group. There was no group by time interaction for the cognitive variables. At the second timepoint, 67% of the rehabilitation group were employed, compared to 33% of the control group.

**Conclusion:**

This first-of-its-kind program met its primary goal of supporting reintegration into the workforce among non-CNS cancer survivors with neuropsychological difficulties, alongside additional positive effects.

## 1 Introduction

The term “cancer survivor” describes a heterogeneous group, encompassing individuals during various stages of disease progression, treatment, and recovery (Doose et al., 2022). Based on this definition, there are an estimated 16.9 million survivors in the US alone (Miller et al., 2019).

Some survivors report that when a round of cancer treatment has been completed and deemed successful, societal, social, and family circles believe they are able to continue life as before the diagnosis (Wells et al., 2022). In reality, however, even cancer survivors who are considered cured or in remission can face a range of obstacles (Öcalan and Üzar-Özçetin, 2023).

Prominent among these is difficulty reentering the workforce. Cancer survivors are more likely to be unemployed than healthy control participants (de Boer et al., 2009), with a reported employment percentage between 41 to 84% (Taskila and Lindbohm, 2007). Furthermore, employed survivors frequently report difficulties in the workplace, including reduced productivity and perceived lack of support for emotional challenges (Fitch and Nicoll, 2019). For example, they reported inability to work full-time, and the need for flexibility in workload, time off for appointments, and increased emotional support from colleagues and employers.

More broadly, neuropsychological changes involving both cognitive and emotional functions underlie many vocational difficulties following cancer (Swain et al., 2020). Such changes, particularly expressed in tasks requiring attention, memory, and executive functions (Cerulla Torrente et al., 2020), have been consistently documented in cancer survivors for decades, and can have substantial effects on functioning and quality of life (Firkins et al., 2020).

Years of multidisciplinary research corroborate pervasive clinical reports of cognitive impairments in patients with non-central nervous system (CNS) cancer in a phenomenon previously called “chemo-fog” or “chemo-brain” (Janelsins et al., 2018). Evidence has since shown that treatments other than chemotherapy are also associated with such impairments (Ehrenstein et al., 2020). Today, the term cancer-related cognitive impairment (CRCI) encompasses direct and indirect implications of cancer itself and its various treatments (Cerulla Torrente et al., 2020).

CRCI is highly prevalent and frequently long-lasting, with impairments reported in up to 30% of patients prior to chemotherapy, 75% of patients during treatment, and 35% of patients over 10 years after completing treatment (Janelsins et al., 2014). Most patients improve with time, but some continue to show persistent cognitive difficulties. Studies in animals (Fleming et al., 2023) and humans (Kesler and Blayney, 2016) indicate systemic and neurological changes consistent with reported cognitive impairments.

Emotional changes can also hinder the return to routine following cancer, both directly and through their effects on cognitive functioning (Emery et al., 2022). Depression, anxiety, and chronic fatigue are particularly common (Feng et al., 2018), as is fear of cancer recurrence (FCR), which has been reported in 78% of survivors and patients (Luigjes-Huizer et al., 2022). Separately and interactively, cognitive and emotional difficulties constitute a significant obstacle for survivors, making these neuropsychological changes a key target for intervention (Sleurs et al., 2022).

Attempts to address neuropsychological changes through interventions for cancer survivors are described both outside and within the vocational context, and generally separate the cognitive and emotional domains. Outside the vocational context, Schuurs and Green (2013) assessed a group-based cognitive rehabilitation intervention. They reported longstanding improvements in cognitive function, as well as reduced perceptions of cognitive impairment and psychosocial distress, improved social functioning and understanding of cognition, and high levels of satisfaction with treatment. Telehealth interventions focusing on psychoeducation and compensation strategy training have also led to self-reported cognitive improvements (Myers et al., 2020). Likewise, a range of psychotherapy interventions have successfully addressed cancer-related emotional changes, for example mindfulness and meditation-based therapies (Goyal et al., 2014) and short-term metacognitive therapy (Cherry et al., 2019). Within the vocational context, Guo et al. (2021) reviewed interventions aimed at facilitating return to work after cancer. They concluded that programs combining multiple types of interventions were the most promising, though there was still room for improvement.

Despite the success of targeted cognitive and emotional interventions and their demonstrated relevance to successful vocational integration, post-cancer vocational rehabilitation programs do not generally incorporate both in a holistic manner. Lamore et al. (2019) noted the unidimensional nature of most vocational rehabilitation programs, and suggested that multi-componential interventions would be more effective. Indeed, while later studies show that post-cancer return-to-work programs are feasible and effective in breast cancer populations in which cognitive decline was not an inclusion criterion (Sheppard et al., 2023), we did not find studies describing holistic vocational rehabilitation programs incorporating interventions for both cognitive and emotional functioning among participants with diverse types of non-CNS cancer. Given the small number of prior studies, their largely unidimensional nature, and their focus on specific populations, the current study addressed the still unmet need to examine multidimensional programs with a significant psychological component, for all survivors of non-CNS cancer. Here, we report the preliminary results of a novel, multi-component post-cancer vocational rehabilitation intervention, based largely on holistic neuropsychological rehabilitation day programs for individuals with acquired brain injury (ABI).

The rationale behind basing a novel rehabilitation program for individuals with CRCI on existing programs for individuals with ABI stems from significant similarities between the two groups, in terms of the underlying neurological changes, reported cognitive and emotional difficulties, and impacts on daily and vocational functioning described above (Nakamura et al., 2024). While CRCI and ABI differ with respect to the source of neurological damage, both are associated with considerable variability in the severity and specific profile of neuropsychological difficulties. Group-based ABI rehabilitation programs are designed to address this variability, facilitating their adaptation for a population with similar, though not identical, characteristics.

The National Institute of Neuropsychological Rehabilitation in Israel (henceforth, “the Institute”) runs several programs based on the holistic bio-psycho-social model of rehabilitation, to reintegrate individuals with acquired CNS damage. The day programs incorporate individual and group interventions targeting emotional, cognitive, functional, interpersonal, and vocational components, including career exploration and experiential training. Intensive curriculums address the specific needs of individual participants with mild, moderate, or severe ABI, as determined in pre-treatment neuropsychological assessments. Programs generally last 10 months for 4–5 days a week, and then offer individually-tailored follow-up for several years. Studies addressing the efficacy of similar vocation-focused community-based neuropsychological rehabilitation interventions largely indicate that they improve general functioning and employment status (Shany-Ur et al., 2020).

The currently examined 6-month program was designed in light of similarities between the neuropsychological deficits reported in CRCI and those associated with mild ABI (Lv et al., 2020). With the aforementioned principles as a framework, the Sulam (“ladder”) program offered immersive vocation-focused rehabilitation to cancer survivors experiencing neuropsychological difficulties. The aim was to apply vast theoretical knowledge and clinical experience, developed and refined for individuals with ABI, to help non-CNS cancer survivors with similar difficulties reintegrate into the workforce. Beyond this shared backbone, the interventions were tailored to the specific cognitive and emotional challenges associated with return to life and work after cancer treatment, such as fertility, fear of a recurring life-threatening illness, and gaps between social perceptions of recovery and actual functioning. While maintaining the intensity (4–5 days a week) and relatively long duration of the 10-month ABI programs, meant to enable significant neuropsychological changes and coping mechanisms that take time to develop, the current program was shortened to 6 months to account for the generally milder cognitive decline and greater employment potential of the CRCI population.

The current study examined Sulam program implementation across the first eight cohorts. Main objectives included initial assessment of feasibility in terms of participant employment status, cognitive functioning, and emotional state. We hypothesized that rehabilitation group participants would improve on all measures following completion of the program, as compared to the control group. The Ethics and Research Committee of the National Institute of Neuropsychological Rehabilitation approved the research, approval number 05/20.

## 2 Methods

### 2.1 Participants

The rehabilitation group comprised 64 individuals (59 female; average age 45.7 years, SD 7.7) who participated in 1 of 8 cohorts of the Sulam holistic vocational rehabilitation day program for non-CNS cancer survivors at the Institute between December 2018 and December 2020. Participants in this group were referred and funded by the Rehabilitation Department of the National Insurance Institute (equivalent to the Social Security Administration in the USA). Under the funding arrangement, participants received a monthly stipend, conditional on completion of a predetermined minimum of weekly hours. The assumption motivating this arrangement is that the program will enable participants, otherwise likely to remain unemployed and require long term government aid, to enter the workforce, receive salaries allowing them to fund themselves independently, and pay taxes for a considerable number of years.

To be included in the program, participants had to have completed treatment for any form of non-CNS cancer at any time in the past, be unemployed (i.e., not participating in any daily activity, paid or unpaid, regardless of other sources of income), express high motivation for employment, show cognitive deficits in a formal neuropsychological assessment (below average on at least one measure), and report post-cancer cognitive decline or functional difficulties. Exclusion criteria were severe cognitive deficits and psychological or emotional states precluding participation, as deemed by a licensed psychologist. Of the entire group, 46 completed the study measures at the second time point, after 3 withdrew from the program, 2 died, and 13 voluntarily withdrew from the study (but not the program) due to COVID-19 related restrictions and challenges.

A control group of 24 (22 female; average age 45.4, SD 7.7) was recruited via social media. We originally planned to recruit controls from the waitlist for program participation. However, due to high demand, the Institute opened cohorts faster and with greater geographical spread than initially expected. This reduced the number of individuals on the list and their wait times. We therefore recruited control participants via social media, resulting in a relatively small group and in non-random allocation between the two groups. Control participants were subject to the same inclusion and exclusion criteria as the rehabilitation group, but did not participate in the rehabilitation program. They were tested at the first time point upon recruitment, and 15 of them were tested once again at a second time point 6 months later, after 9 voluntarily withdrew from the study. There were no significant differences between participants who remained in the study and those who withdrew on any of the personal, cognitive, or emotional variables (all *p*s ns).

### 2.2 Measures

All measures were administered in Hebrew.

### 2.2.1 Cognitive measures

We assessed cognitive functioning using a validated, computerized test battery (NeuroTrax®; Neurotrax Corp., Modiin, Israel) that detects mild cognitive impairments (de Oliveira and Brucki, 2014). The battery addresses seven cognitive domains: memory, executive function, visual–spatial processing, verbal function, attention, information processing, motor skills. It provides age-and education-adjusted index scores for each domain alongside a global score. All scores are fit to a standardized scale, with a mean of 100 and standard deviation of 15. Scores lower than 85 indicate impairment. Test–retest reliability has been found accurate (Boussi-Gross et al., 2015). Here, we examined global score, as well as memory, executive function, and attention, as impairments in these areas are most commonly reported in non-CNS CRCI and most often demonstrated in the literature. All assessments were conducted in person or remotely, in accordance with participant preference and COVID-19 restrictions. The manner of data collection did not differ between the two timepoints, nor between the rehabilitation and control groups.

#### 2.2.2 Emotional measures

Emotional measures included the Beck Depression Inventory (BDI-2) (Beck et al., 1996), a brief, self-report questionnaire designed to measure the severity of depression symptomatology; the Hospital Anxiety and Depression Scale (HADS) (Zigmond and Snaith, 1983), administered as a self-report questionnaire; and a questionnaire developed by the program staff to assess fear of cancer recurrence (FCR), in which three questions were rated on 5-point scale with higher scores representing greater fear: (1) To what extent are you afraid that your cancer will return? (2) To what extent does this fear affect your daily functioning? (3) To what extent does this fear affect your plans for the future?

#### 2.2.3 Employment measure

Employment status was a dichotomous measure indicating whether participants were unemployed or either employed or participating in a structured education or training program.

### 2.3 Program description

**Figure 1:**
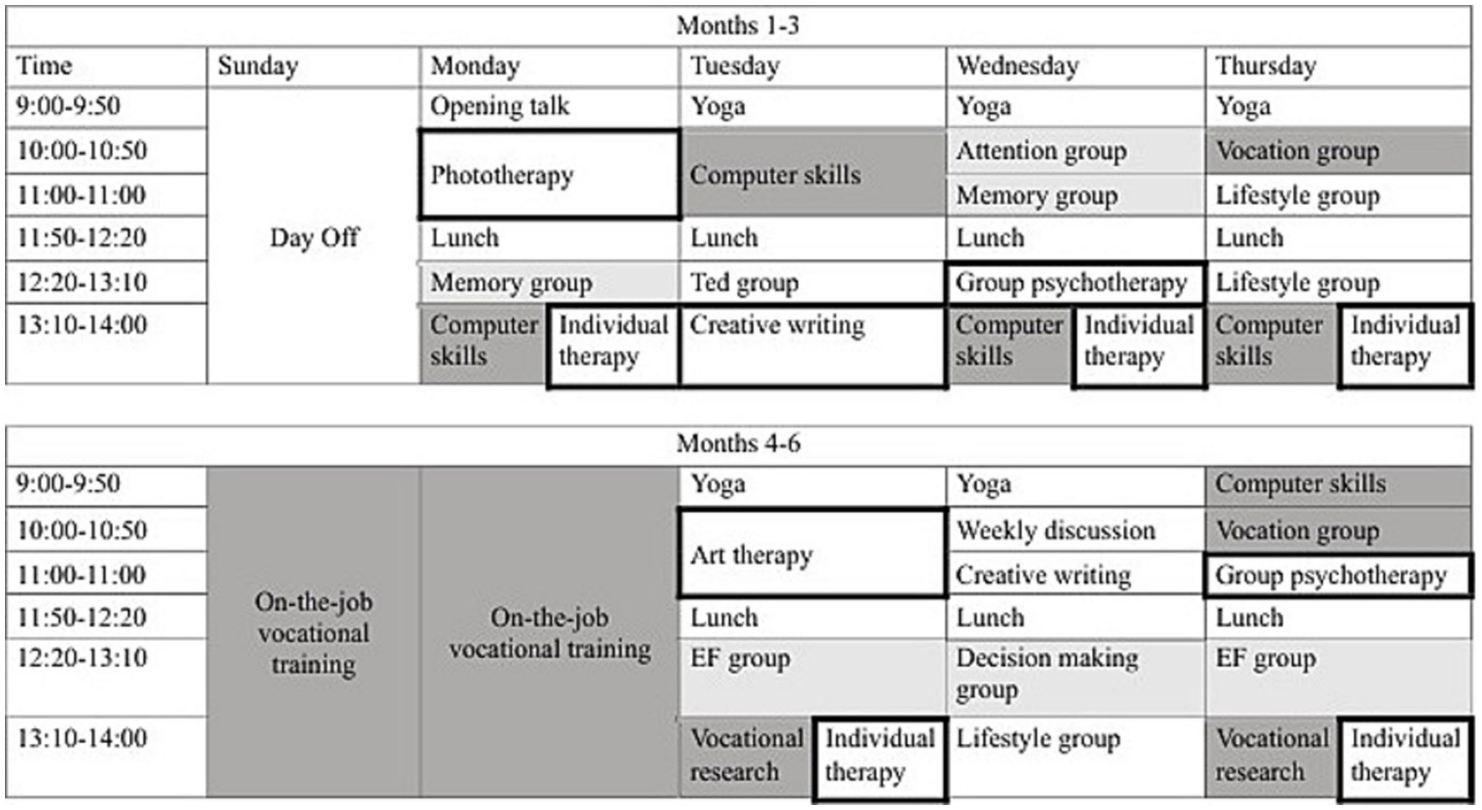
Sample schedule for the Sulam program. Dark grey, vocational components; light grey, cognitive components; bold frame, emotional components; EF, executive functions.

The 6-month program comprised two 3-month stages, each with a structured weekly schedule. There were 6–12 participants in each of 8 cohorts. The first stage included individual and group sessions 4 days a week, addressing cognitive functioning (attention, memory, and executive function groups; psychoeducation groups), emotional functioning (individual psychotherapy, group psychotherapy, psychodrama, art therapy, creative writing), vocational preparation (individual and group vocational counseling, technology skills, job research), and healthy lifestyle (e.g., self-management and daily functioning, pain coping strategies, yoga and mindfulness training, nutrition). Schedules were designed and supervised by program coordinators, who were practicing neuropsychologists. The second stage included 3 days a week of sessions and 2 days of guided reintegration into individually-tailored trial employment settings. Figure 1 presents a sample schedule. Many participants stayed on in these settings as paid employees following program completion. Due to the COVID-19 pandemic, parts of the program were administered remotely in some cohorts.

### 2.4 Study procedure

#### 2.4.1 Rehabilitation group

The Institute conducted comprehensive neuropsychological assessments as part of the screening process for program participation, following referral by the National Insurance Institute. Suitable individuals were approved for funding and entered the program. During the intake session, program staff described the study goals and procedure and asked program participants whether they would be interested in enrolling. Interested individuals underwent a structured informed consent process and signed consent forms. The study measures were among those included in the initial assessment, which served as the first time point (T1). The second round of data (T2) was collected upon program completion by Institute staff members who were not involved in administration of program interventions.

#### 2.4.2 Control group

Potential participants responded to social media posts and advertisements by phone. During the initial call, a staff member described the study and assessed exclusion and inclusion criteria. Those who agreed underwent the informed consent process and signed consent forms and then underwent the same tests as the rehabilitation group. They were also told about the program itself and had the option of entering the screening process to join a cohort upon study completion. Institute staff collected the study measures in control group participants at 2 time points, 6 months apart. Each session lasted approximately 1 h. Control participants received gift cards ($25 for each session). The decision to compensate control participants was made to address the specific recruitment challenges encountered with this group, which lacked direct affiliation with the Institute, in contrast to the intervention group, which was recruited from among program participants. This difference in compensation could have influenced participation motivation and group composition, which should be considered when interpreting the findings.

### 2.5 Statistical analysis

To test the research hypotheses, we used two-way mixed-design analyses of variance (ANOVAs) with time (T1, T2) as a within-subject variable and group (control, rehabilitation) as a between-subject variable. The dependent variables included the NeuroTrax scores (global score, memory, executive function, attention) and the emotional variables (BDI, HADS, FCR). When interactions were found, simple analyses were conducted. We used McNemar’s test to compare employment rates between the two timepoints for each group separately.

## 3 Results

### 3.1 Preliminary analyses

The two groups did not differ significantly in sex [ $\chi^{2}$ (1) = 0.57, *p* = 0.452] or age [*t* (61) = 0.49, *p* = 0.626] (Table 1). *T*-tests revealed no significant between-group differences in any of the NeuroTrax scores or emotional variables at the first timepoint (largest *t* = 1.29, largest *p* = 0.203). Both groups were heterogeneous in terms of cancer type, location, and stage, but approximately 40% (in both groups) had breast cancer at various stages and the remaining 60% were diverse with no one type, location, or stage constituting a significant percentage.

**Table 1:** Sex and age by study group.

	Control	Rehabilitation	*Statistic*	*p*
Sex, *n* (%)			$\chi^{2}$ (1) = 0.57	0.452
Male	0 (0.0%)	2 (3.9%)		
Female	14 (100.0%)	49 (96.1%)		
Age, *M (SD)*	47.00 (5.82)	45.82 (7.81)	*t* (61) = 0.49	0.626

### 3.2 Research hypotheses

**Figure 2:**
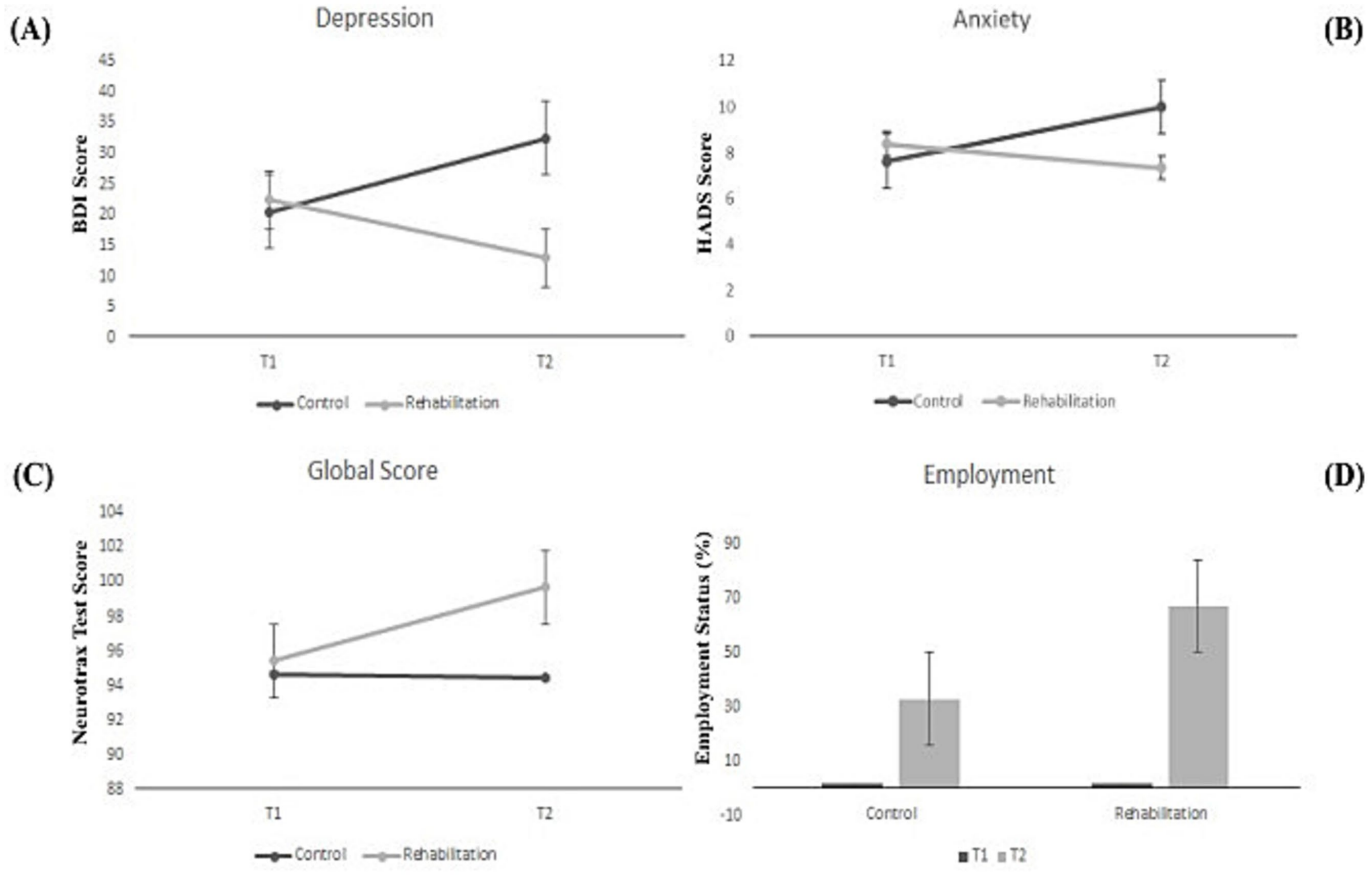
Results of between-group comparisons for **(A)** the Beck Depression Inventory, **(B)** the Hospital Anxiety and Depression Scale, **(C)** Neurotrax Global Score, and **(D)** employment status.

Table 2 presents the descriptive statistics for both groups and results of the between-group comparisons, some of which are shown in Figure 2. All participants were unemployed at the first timepoint. At the second timepoint, 67% of the rehabilitation group were employed, compared to 33% of the control group. McNemar tests indicated that employment percentage increased significantly in the rehabilitation group but not in the control group.

**Table 2:** NeuroTrax scores, emotional variables, and employment status in each group and timepoint, and results of between-group comparisons.

Variables	Study Group	*N*	T1		T2		Simple Effect	ANOVA Results					
								Time			Time X Group		
			*M*	*SD*	*M*	*SD*		*F*	*P*	*η* ^2^	*F*	*p*	*η* ^2^
Global Score	Control	12	94.68	14.63	94.43	19.56	−0.26	2.49	0.121	0.04	3.17	0.081	0.06
	Rehabilitation	43	95.44	8.22	99.69	8.90	4.24***						
Memory	Control	12	91.56	19.23	96.58	20.18	5.02	3.72	0.059	0.07	0.47	0.497	0.01
	Rehabilitation	43	96.47	12.93	98.85	13.59	2.39						
Executive Function	Control	12	91.90	15.87	97.63	17.65	5.73+	12.33	0.001	0.19	0.01	0.917	0.00
	Rehabilitation	42	95.71	9.28	101.79	11.26	6.08***						
Attention	Control	12	86.89	14.72	88.05	22.65	1.16	2.63	0.111	0.05	1.22	0.275	0.02
	Rehabilitation	43	91.61	11.49	97.68	11.70	6.07**						
Beck Depression Inventory	Control	8	20.50	2.12	32.50	19.09	12.00	0.11	0.745	0.01	7.19	0.015	0.27
	Rehabilitation	31	22.37	9.23	13.00	9.06	−9.37**						
Hospital Anxiety and Depression Scale	Control	8	7.63	4.17	10.00	4.41	2.38+	0.90	0.349	0.02	5.80	0.021	0.14
	Rehabilitation	31	8.39	3.89	7.35	4.66	−1.03						
Fear of Cancer Recurrence	Control	6	2.72	0.77	3.39	0.95	0.67+	0.47	0.500	0.02	7.28	0.012	0.23
	Rehabilitation	21	2.86	0.87	2.46	0.77	−0.40*						
Employment	Control	9	*n* = 0 (0%)		*n* = 3 (33%)				0.250^a^				
	Rehabilitation	46	*n* = 0 (0%)		*n* = 31 (67%)				<.001^a^				

+*p* < 0.10, *p < 0.05, **p < 0.01, ***p < 0.001.

a Significance of the change in employment rate calculated using McNemar’s test.

ANOVA results indicated significant Time x Group interactions for each of the emotional variables: BDI (*p* = 0.015), HADS (*p* = 0.021), and FCR (*p* = 0.012). Further analyses showed significant decreases in depression and fear of cancer recurrence at the second timepoint in the rehabilitation group but not in the control group. The Time x Group interaction in HADS stemmed from an increase in the control group compared to a decrease in the rehabilitation group, although neither of these within-group effects was significant (i.e., neither group showed a significant change in HADS from the first timepoint to the second).

As seen in Table 2, average depression levels in the control group were in the moderate range at the first timepoint and in the severe range at the second timepoint. In contrast, average depression in the rehabilitation group went from moderate at the first timepoint to mild at the second timepoint (HADS ranges: 0–9 minimal depression, 10–18 mild, 19–29 moderate, 30–63 severe).

ANOVA results for the NeuroTrax scores indicated no significant Time x Group interactions, though global score results were marginally significant (*p* = 0.081) based on a significant increase between the timepoints in the rehabilitation group but not in the control group. The main effect of time was significant for executive functions (*p* = 0.001) and marginally significant for memory (*p* = 0.059), indicating an overall increase in both cases.

## 4 Discussion

We examined a holistic, community-based neuropsychological rehabilitation program for cancer survivors aiming to return to the workforce. The eight included cohorts successfully reached completion, with minimal withdrawal from the intervention (independent of study withdrawal). Despite the notable intensity and duration, the vast majority of participants completed the program. The results indicate that the program is feasible and largely fulfilled its primary aim, with the rehabilitation group showing improvement on most of the study measures.

The percentage of employed program participants rose from zero to 67%, while the increase in the control group was smaller. Effects on emotional variables were particularly significant, as the rehabilitation group showed reductions in depression, anxiety, and fear of cancer recurrence, while these measures increased in the control group. Program participants also showed significant increases in executive functions, attention, and global score. Between-group differences did not, however, reach significance, as the control group also tended to have higher scores at the second time point. This suggests that improvements in the rehabilitation group could not be attributed to program participation alone.

### 4.1 Supporting reintegration into employment

Beyond financial and social implications, return to work following a significant illness is associated with improved perceived quality of life and psychological well-being, with survivors and their families deeming it an important goal (Fitch and Nicoll, 2019). In accordance, reintegration was the primary aim of the currently described program. This aim was achieved, with two-thirds of the rehabilitation group holding a job at the second time point, compared to one-third of controls. The jobs they entered were diverse, in accordance with their education, experience, and functional status, for example positions in law, management, and back office.

These findings align with previous reports of improvement following vocation-centered interventions that were specifically designed to address CRCI and return to work in cancer survivors. Reporting on a program involving individual sessions with an occupational therapist, Sheppard et al. (2020) showed that 67% of participants had improved capacity to work and 87% had better work status upon completion. Sheppard et al. (2023) later expanded on these findings, reporting high levels of commitment to and satisfaction with the “Beyond Cancer” program for breast cancer survivors with cognitive decline.

Unlike the programs in Sheppard et al. (2020, 2023), the current program and study addressed a subset of survivors with different forms of non-CNS cancer who experienced longer-term difficulties returning to work and were specifically concerned with cognitive decline. The currently described approach, which augmented vocational interventions with cognitive rehabilitation and interventions specifically targeting emotional issues, produced promising results similar to those described in prior work, in a population with broader difficulties.

### 4.2 Cognitive functioning

The Sulam program addressed cognition as both a direct target and a strong correlate of vocational functioning. Contrary to expectations, support for cognitive improvements specific to the rehabilitation group was not clear. The global measure showed a marginally significant interaction stemming from a significant improvement in the rehabilitation group that was not seen in controls. However, this pattern was not repeated with respect to memory, executive functioning, or attention. Across groups, the general direction was improvement in cognitive functioning over time, with significant improvement in executive functioning and marginally significant improvement in memory.

Prior research examining how post-cancer rehabilitation affects cognition has produced mixed findings. Interventions based on psychoeducation and cognitive compensation have led to self-reported improvements in cognitive functioning (Myers et al., 2020) but these have not always been qualified by measurable changes in neuropsychological evaluations (Cheng et al., 2022). This suggests that interventions acknowledging and addressing cognitive difficulties have value for individuals with CRCI, even in the absence of measurable improvements. In the current study, cognitive functioning was largely addressed as a means for supporting vocational skills. In this context, the improvements in vocational status among participants support the inclusion of cognitive interventions in multifaceted vocational rehabilitation programs. It is possible that a different division between cognitive and emotional intervention components, or an increase in cognitive components, would have resulted in measurable cognitive improvement. This can be addressed in future work.

Across groups, our sample showed improvements on cognitive measures at the second time point, likely reflecting spontaneous changes in neural and cognitive functioning over time. This is in line with work showing that the percentage of cancer survivors who experience cognitive difficulties decreases over time (Sousa et al., 2020). It should also be noted, however, that despite their substantial effects on daily functioning, cognitive difficulties associated with non-CNS cancer are often mild relative to more serious neurological conditions. This could lead to a ceiling effect when they are measured using test batteries designed to address a broader spectrum of deficits. Measures more sensitive to mild impairments could more accurately indicate post-cancer changes in cognition.

### 4.3 Emotional functioning

Program interventions targeting emotional functioning, including individual and group psychotherapy, yoga, art therapy, and social and recreational activities, were tailored to address the high levels of anxiety and depression characteristic of the participant population. Beyond the direct importance of emotional well-being, this aim stemmed from well-documented ties between emotional and vocational functioning (Gedikli et al., 2023).

Program participants showed significant declines in depression and fear of cancer recurrence, while control participants did not. Additionally, there was a tendency toward increased anxiety in the control group that did not occur in the rehabilitation group. Extending similar findings on individual psychotherapy and emotional interventions in the context of broader post-cancer rehabilitation (Olsson Möller et al., 2019), these results suggest that the program reduced depression and fear of cancer recurrence while moderating increases in anxiety. These findings should be further explored in a randomized controlled trial to address effectiveness.

### 4.4 Study limitations

Like much research examining the feasibility or effectiveness of therapeutic interventions, the current study was associated with methodological and ethical challenges. Due to the group size limitations and the large difference between the group sizes, we might have missed significant effects. We did not perform an *a priori* power analysis, but it is likely that the study lacks power. The non-randomized sampling method, in which control participants were recruited from social media and chose not to participate in the program, might also have created a selection bias that could explain the between-group differences in return to work. Specifically, it stands to reason that individuals who chose not to participate in a vocational rehabilitation program would be less motivated to return to work than those who chose to participate. This could be evaluated in a randomized control design, which would be crucial to a rigorous evaluation of intervention efficacy in general.

The size and nature of our sample also precluded calculation of a meaningful internal reliability measure for the questions we used to assess fear of cancer recurrence. While we chose to include the measure in our analysis, given the face validity of the questions and the inherently subjective nature of FCR, we acknowledge that use of a validated tool would have been preferable. Additionally, the current study did not specifically assess pre-intervention similarities in motivation to return to work, which should be considered in future research.

Another methodological difficulty in evaluating multi-componential clinical interventions involves understanding the separate and interactive contributions of different components. With the current findings constituting a preliminary indication of the feasibility of the Sulam program, the pool of potential participants and the time span during which they can be studied will grow. Larger sample sizes and longitudinal designs will make it possible to address some of the methodological challenges inherent in this type of evaluation, for example through statistical models examining the relationships between participant characteristics, interventions components, and specific outcomes. A design based on Klaver et al. (2020), in which full and partial programs are compared, and additional outcome measures, such as adherence and self-reported participant satisfaction, would be meaningful additions to future research.

There were additional limitations specific to the current sample. We included a significantly larger number of female participants, reflecting a similar bias in referrals from the social security system. It was beyond the scope of the current work to analyze the sources of this bias, which should be addressed in future research to ensure that advances in post-cancer vocational rehabilitation are accessible and tailored to the needs of all survivors. Also notable is the fact that much of the current research was conducted during the global COVID-19 pandemic, such that some cohorts attended some interventions remotely. The pandemic also had implications on participant adherence, limiting the possibility to conduct a rigorous and generalizable feasibility examination. Feasibility examinations were further limited by the failure to collect sufficient, structured feedback from participants and other stakeholders (e.g., referring parties), which should be corrected in future research.

Despite these limitations, which should be remedied in further research, we believe there is value in introducing the program and showing the initial findings.

### 4.5 Clinical implications

This study presents the first vocation-based rehabilitation program for non-CNS cancer survivors with cognitive complains, based on the holistic bio-psycho-social model of ABI rehabilitation. It demonstrates the value of this framework in developing vocational rehabilitation interventions for cancer survivors with neuropsychological deficits. As such, it extends support for developing and examining such programs, aimed at integrating cancer survivors into the workforce while addressing the emotional and cognitive difficulties affecting their vocational capacities.

## 5 Conclusion

A novel vocation-focused rehabilitation program, based on theoretical knowledge and clinical experience from the field of ABI rehabilitation, offered diverse interventions tailored to the specific cognitive and emotional challenges associated with return to life and work after cancer treatment. The findings, which indicated rehabilitation-related improvements in employment status as well as emotional and cognitive functioning, support continued development of this and similar interventions.

A foundational principle taken from community-based vocational rehabilitation for individuals with ABI is that the whole is greater than the sum of its parts. Namely, there is significant value in the holistic approach, and interventions acknowledging and addressing a specific component can contribute to overall rehabilitation even in the absence of measurable gains in that component. Specifically with respect to CRCI in survivors of non-CNS cancer, there is still much to be learned by examining vocational rehabilitation programs that address emotional and cognitive impairments and integrate interventions targeting various aspects of functioning and participation.

## Data Availability

The raw data supporting the conclusions of this article will be made available by the authors, without undue reservation.
